# Case Report: Bilateral Renal Cell Carcinoma With Different Histological and Morphological Features, Clear Cell and Cystic Thyroid-Like Follicular Subtype

**DOI:** 10.3389/fonc.2021.659706

**Published:** 2021-04-26

**Authors:** Jinsong Ni, Ni Cui, Yanfang Wang, Jixuan Liu

**Affiliations:** ^1^ Department of Pathology, The First Hospital of Jilin University, Changchun, China; ^2^ Department of General Surgery, China-Japan Union Hospital of Jilin University, Changchun, China; ^3^ Department of Pathology, Qianwei Hospital of Jilin Province, Changchun, China

**Keywords:** bilateral renal cancer, cyst, pathological diagnosis, imaging diagnosis, surgical treatment, thyroid-like follicular carcinoma of kidney

## Abstract

Thyroid-like follicular renal cell carcinoma is a rare subtype of renal cell carcinoma that has only been recently recognized, as most cases involve a solid tumor in one kidney. In this study, we report a rare case of bilateral renal cell carcinoma wherein the tumor in the left kidney was diagnosed as clear cell carcinoma, while the tumor in right kidney as thyroid-like follicular renal cell carcinoma. The difference between this case and the ones described in previous reports is that thyroid-like follicular renal cell carcinoma showed cystic changes on imaging. This suggests that when renal cystic lesions are encountered, we should consider the possibility of such rare tumors.

## Introduction

Thyroid-like follicular renal cell carcinoma is a rare tumor, which is not included in the 2016 WHO Classification of Tumours of the Urinary System and Male Genital Organs (1). In recent years, increasing numbers of reports on this tumor type have been published (2–5), which suggests the increase in prevalence of this subtype of kidney tumor. Most of the reported thyroid follicular renal cell carcinomas are solid tumors, and only one case with cystic changes on imaging has been reported (6). In patients with this tumor, simple renal cysts and multilocular cystic renal neoplasm of low malignant potential require differential diagnosis.

Bilateral renal tumors are rare, as they have been reported to be present in 1–4% of renal cancer patients (7). Surgery is the conventional treatment, but balancing oncological efficacy and preservation of renal function is challenging. In patients with bilateral renal tumors, four different surgical approaches are typically available: radical nephrectomy followed by contralateral partial nephrectomy, partial nephrectomy followed by contralateral radical nephrectomy, bilateral partial nephrectomy, and bilateral radical nephrectomy (8, 9). Some guidelines and experts suggest that bilateral partial nephrectomy should be preferred for bilateral renal tumors. However, further research is warranted for the treatment of tumors with uncertain malignant potential or degree of malignancy.

## Case Description

**Figure 1:**
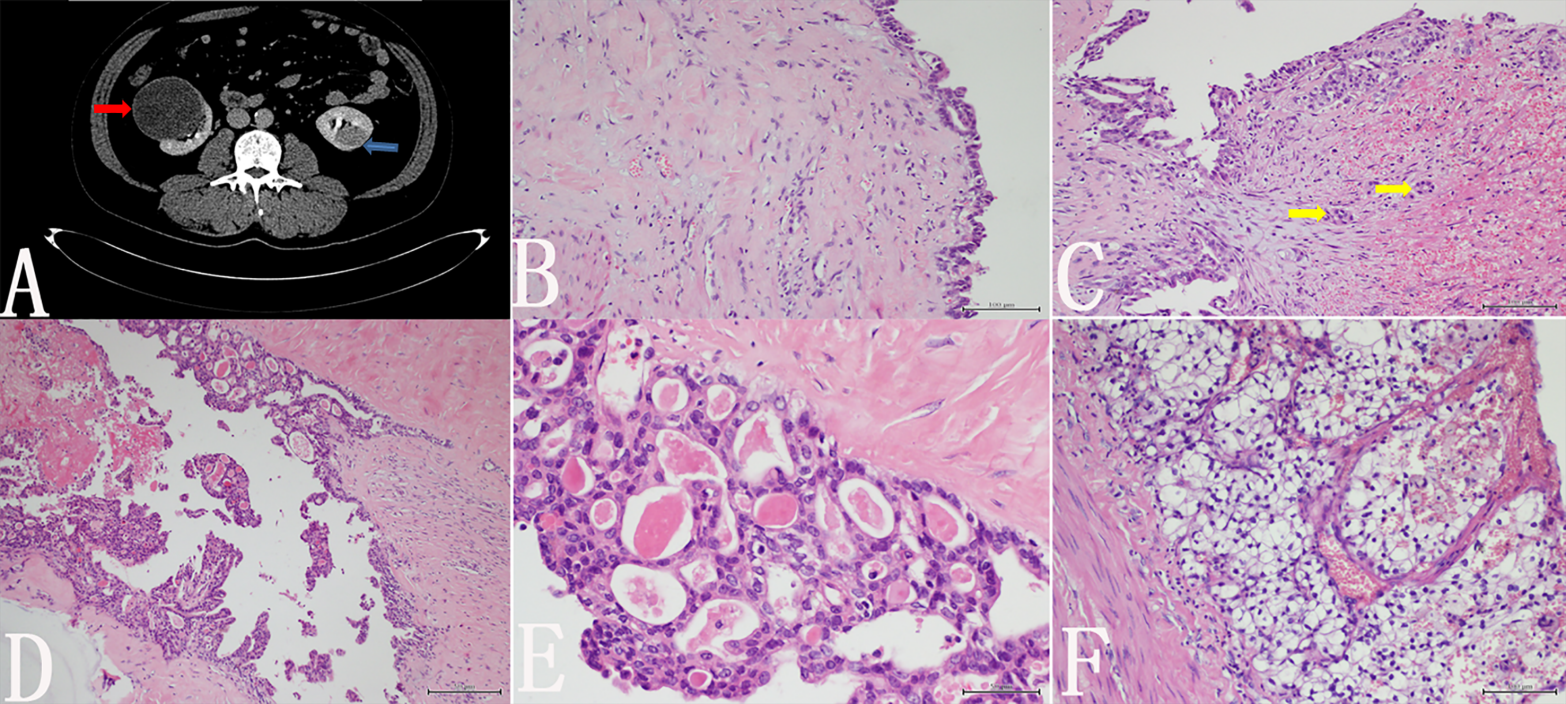
**(A)** Abdominal computed tomography revealed a relatively homogeneously enhancing mass in the left kidney (blue arrow) and a cystic lesion in the right kidney (red arrow). **(B)** Histology showed cells arranged in a single or multiple layers, which could be seen in some areas of the cyst wall. Cellular atypia, nucleoli, and mitosis were not obvious. **(C)** Necrosis and hemorrhage are visible in another area, and some suspicious infiltrating glands were seen in the fibrous stroma (yellow arrow). **(D)** In the postoperative pathological examination, we observed follicular architecture with micro- and macrofollicles containing eosinophilic secretions or colloid-like material in the cyst wall of the right kidney. **(E)** Follicular cells contained moderate eosinophilic cytoplasm and round nuclei. **(F)** The left renal tumor is a typical clear cell renal cell carcinoma.

**Figure 2:**
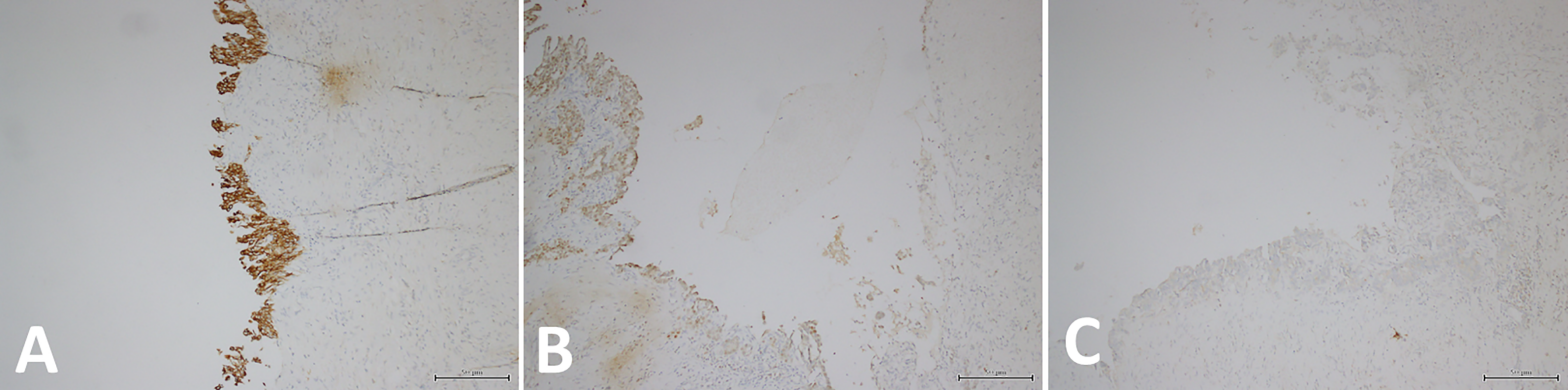
**(A)** Immunohistochemistry revealed that the tumor cells of the right kidney tumor were CK7-positive. **(B)** Immunohistochemistry also revealed that the tumor cells of the right kidney tumor were PAX2-positive. **(C)** Immunohistochemistry showed that the tumor cells of right kidney tumor were TTF1-negative.

The patient is a 47-year-old male. He was found to have space-occupying lesions on both kidneys during routine physical examination and presented to our hospital for diagnosis and treatment. Laboratory test results including those for thyroid function were within normal ranges. Abdominal computed tomography revealed a relatively homogeneously enhancing mass in the lower pole of the left kidney (4 cm in its greatest dimension) and a cystic lesion in the right kidney (**Figure 1A**). Part of the wall of the right renal cyst was surgically removed, and intraoperative pathological examination was performed. Histology showed cells arranged in a single or multiple layers, which could be seen in some areas of the cyst wall. Cellular atypia, nucleoli, and mitosis were not obvious (**Figure 1B**). Obvious necrosis and hemorrhage could be seen in another area, and the fibrous tissue in the cyst wall was perforated; some suspicious infiltrating glands could also be seen in the fibrous stroma (**Figure 1C**). We suspected cystic renal cell carcinoma. To preserve some kidney function, the patient underwent partial nephrectomy of the right kidney with nephron preservation and left radical nephrectomy. According to the postoperative pathology examination, we observed follicular architecture with micro- and macrofollicles containing eosinophilic secretions or colloid-like material in another area of the right renal cyst wall (**Figure 1D**). The follicular cells contained moderate eosinophilic cytoplasm and round nuclei (**Figure 1E**). The left renal tumor was a typical clear cell renal cell carcinoma (**Figure 1F**). Immunohistochemistry revealed that the tumor cells of the right kidney tumor were positive for cytokeratin 7 (CK7) (**Figure 2A**) and paired Box 2 (PAX2) (**Figure 2B**) but were negative for transcription termination factor 1 (TTF1) (**Figure 2C**), thyroglobulin (TG), and synaptophysin (Syn). There was no evidence of a mass in the thyroid gland or enlargement of regional lymph nodes on ultrasonography of the neck. Therefore, the right renal tumor was finally diagnosed as thyroid-like follicular renal cell carcinoma with cystic changes, while the left kidney tumor was diagnosed as clear cell renal cell carcinoma. The patient did not receive postoperative adjuvant treatment. He was discharged five days after the procedure, and his serum creatinine level was 110 µmol/L at discharge. At the time of this writing, the patient is 10 months post-treatment with no clinical or histological evidence of recurrence.

## Discussion

Thyroid-like follicular carcinoma of the kidney is a rare entity, as only approximately 40 cases have been reported in the literature (10). Most cases reported in the literature are solid tumors, and cystic changes may occur in some tumors. However, in rare cases, the tumor will exhibit imaging findings similar to those of simple renal cysts, which may lead to misdiagnosis. Jenny J Ko et al. reported a case of thyroid-like follicular renal cell carcinoma with bone metastasis wherein the patient’s CT showed renal cystic changes (10). If bone metastasis is not present, the patient will not likely receive treatment. This is similar to our case, where imaging of our patient revealed renal carcinoma on one side and cystic changes on the other. If a patient is only observed to have a cyst on one kidney during a physical examination, it is unlikely that the patient will receive sufficient attention if he or she is asymptomatic. This prompts us to stay vigilant, as kidney cysts may also appear malignant.

Surgery is the preferred treatment for bilateral renal cell carcinoma, and some studies suggest that bilateral and unilateral renal cancer show similar patterns of progression and prognosis (11). To preserve renal function, partial removal of the kidney is a reasonable option. Surgical methods include radical nephrectomy in cases of large or multiple tumors, partial nephrectomy for the contralateral kidney with low tumor burden, or bilateral partial nephrectomy for bilateral small tumors (12). Considering the importance of kidney preservation, we surgically removed part of the right kidney. There was no evidence of recurrence or metastasis during the 10-month follow-up after surgery.

Most kidney cysts are simple, but hemorrhage may still occur in these cysts. As hemorrhagic cysts resolve, they develop residual calcification in a central pattern or within the cyst wall that becomes thickened and develops septae. At that point, the cyst becomes multilocular or multilobular and essentially acquires features of a complex cyst (13). This type of cyst should be distinguished from cystic renal cell carcinoma and other cystic malignancies. However, due to bleeding and necrosis, the tumor cells on the inner wall of the cyst are often shed into the cyst cavity. For intraoperative pathologic diagnosis, surgeons often remove part of the cyst wall. When the pathologist is unable to observe the epithelial cells of the more severe neoplastic cyst wall, misdiagnosis may occur. Therefore, for renal cysts, especially complex cysts or cysts with significantly thickened walls, complete removal of the cyst wall may be a more reasonable treatment. In our case, the cyst wall was accompanied by obvious necrosis and bleeding, and most of the cyst wall epithelium had already disappeared. During surgery, we did not observe all the epithelial components of the tumor. However, after surgery, sampling of the entire cyst wall revealed a more typical thyroid-like renal cell carcinoma. This reinforces the importance of sampling all renal cyst lesions.

The most important differential diagnosis of renal thyroid-like renal cell carcinoma is renal metastasis of thyroid cancer or metastatic struma ovarii, since these three tumor types have similar histological manifestations (14). Detection of TG and TTF1 may play a role in the differential diagnosis because primary thyroid cancer and metastatic struma ovarii often express these two proteins (15). Thyroid ultrasound or thyroid CT undoubtedly provides the most direct evidence of a primary thyroid tumor. Our case is a male patient, immunohistochemical staining for TTF1 was negative, and ultrasound of the thyroid did not reveal a lesion. These findings suggest a diagnosis of primary thyroid-like renal cell carcinoma. In view of the extremely low incidence of thyroid-like renal cell carcinoma, when a tumor with a similar morphology is found in the kidney, a secondary tumor must be excluded.

Familial renal cell carcinoma is usually inherited by autosomal dominance, although inherited RCC may present without a relevant family history. Young onset age and bilateral/multicentric tumor are recognized characteristics, which should prompt molecular genetic analysis (16). Our patient had no history of familial renal cell carcinoma. Unfortunately, because of the high cost of testing, patients refused to carry out genetic testing. Further molecular genetic studies are needed for this bilateral renal cell carcinoma with special histological subtypes.

## Conclusion

In this study, we reported a rare case of bilateral renal tumors, with clear cell carcinoma on the left side and thyroid-like renal cell carcinoma on the right side. Specifically, the thyroid-like renal cell carcinoma showed cyst-like changes. This suggests that we should be cautious in the diagnosis of renal cysts based on imaging and should consider the possibility of this rare tumor type when evaluating benign or malignant lesions of complex kidney cysts.

## Data Availability Statement

The original contributions presented in the study are included in the article/supplementary material. Further inquiries can be directed to the corresponding author.

## Ethics Statement

Written informed consent was obtained from the individual(s) for the publication of any potentially identifiable images or data included in this article.

## Author Contributions 

JN and NC were involved in drafting and final revision of the manuscript. JL provided imaging and clinical data, and participated in the editing of the manuscript. YW proofread the final text and revised part of the content. All authors contributed to the article and approved the submitted version.

## Conflict of Interest

The authors declare that the research was conducted in the absence of any commercial or financial relationships that could be construed as a potential conflict of interest.
